# Biodegradation and nutrients removal from greywater by an integrated fixed-film activated sludge (IFAS) in different organic loadings rates

**DOI:** 10.1186/s13568-017-0532-9

**Published:** 2018-01-08

**Authors:** Hadi Eslami, Mohammad Hassan Ehrampoush, Hossein Falahzadeh, Parvaneh Talebi Hematabadi, Rasoul Khosravi, Arash Dalvand, Abbas Esmaeili, Mahmoud Taghavi, Ali Asghar Ebrahimi

**Affiliations:** 1 Department of Environmental Health Engineering, School of Health, Rafsanjan University of Medical Science, Rafsanjan, Iran; 2 Environmental Science and Technology Research Center, Department of Environmental Health Engineering, Shahid Sadoughi University of Medical Sciences, Yazd, Iran; 3 Department of Biostatistics and Epidemiology, Shahid Sadoughi University of Medical Sciences, Yazd, Iran; 4 Social Determinants of Health Research Center, Department of Environmental Health Engineering, School of Health, Birjand University of Medical Sciences, Birjand, Iran; 5 Department of Environmental Health Engineering, Gonabad University of Medical Sciences, Gonabad, Iran

**Keywords:** Greywater, Biodegradation, Integrated fixed-film activated sludge (IFAS), Removal efficiency, Organic loading rate (OLR)

## Abstract

In this study, the efficiency of Integrated Fixed-film Activated Sludge (IFAS) system in synthetic greywater treatment and nutrients removal was studied in duration of 105 days according to different Organic Loadings Rates (OLRs). The study was operated in pilot-scale and OLRs of 0.11–1.3 gCOD/L.d. Scanning Electron Microscope (SEM) image showed that the biofilm with a proper thickness was formed on IFAS reactor’s media. The results indicated that the best removal efficiency of BOD_5_, COD, and TSS were 85.24, 92.52 and 90.21%, respectively, in an organic loading of 0.44 gCOD/L.d. Then, with the OLR increased, the removal efficiencies of BOD_5_, COD, and TSS increased as long as the organic loading reached 0.44 gCOD/L.d. But with the OLR increased more, the removal efficiency of these parameters decreased. The ANOVA statistical test results showed that the mean difference of removal efficiency in organic loadings for BOD_5_ (p ≤ 0.001) and COD (p = 0.003) was significant, while it was insignificant for TSS (p = 0.23). The best removal efficiencies of Total Nitrogen (TN) and Total Phosphorus (TP) were 89.60 and 86.67%, respectively, which were obtained at an OLR of 0.44 gCOD/L.d. By increasing OLR up to 0.44 gCOD/L.d, removal efficiencies of TN and TP increased, while the removal efficiency decreased with the OLR increased more, and this difference was statistically significant (p = 0.021). Finally, the results showed that the IFAS system provided a proper efficiency in treatment of the synthetic greywater and it could be used in a full scale.

## Introduction

Nowadays, the shortage of water resources, growing population and increasing water demand, inappropriate distribution of water resources, mismanagement of the available water, and climate changes have steered most of the countries toward the research for new water resources (Abdel-Shafy and El-Khateeb 2013; Khosravi et al. 2017; Masi et al. 2010; Shahi et al. 2013). One of the ways for compensation of water resources is the reuse of effluent from wastewater (Hourlier et al. 2010), and for the restricted water resources, reuse of domestic wastewater is an important research field in global studies (Ebrahimi et al. 2018; Eslami et al. 2018; Finley et al. 2009).

In general, domestic wastewater includes both greywater and black water (Friedler 2004). The former contains the domestic produced effluent in places, such as the kitchen, showers, baths, wash basins, and laundry. However, the latter is an effluent from toilet (Sanchez et al. 2010). The greywater contains about 70% of the drinkable water or 60–70% of the domestic produced wastewater (Friedler 2004). Per capita production of greywater is 15–55 L per person and varies from 90 to 120 L in a day (Pidou et al. 2007). The greywater specifications are very different depending on the quality of water and type of household activities (Leal et al. 2011). In some studies, the ranges of the various parameters have been reported as follows: 47–466 mg/L for BOD_5_, 100–700 mg/L for COD, 25–183 mg/L for suspended solids, and 29–375 NTU for turbidity (Hourlier et al. 2010; Li et al. 2009). The mean BOD_5_/COD ratio for the greywater has been reported 0.45 ± 0.13 which shows its proper biodegradability potential (Leal et al. 2011). Upon separation of greywater from sewage system, the costs associated with wastewater compound collection network can be saved (Hourlier et al. 2010). Moreover, the greywater is less contaminated as compared to the black water and is suitable for reuse (Gross et al. 2007).With proper treatment of the greywater, the effluent can be used for irrigation, flash tank at homes’ toilet, and other uses (Abdel-Kader 2013).

The various processes used for the treatment of greywater include natural purification systems, such as wetlands (Gross et al. 2014), physical and biological methods, such as filtration (Katukiza et al. 2014a, b), Rotating Biological Contactors (RBC) (Friedler et al. 2005), Up-flow Anaerobic Sludge Blanket (UASB) (Elmitwalli and Otterpohl 2007), Sequencing Batch Reactors (SBR) (Lamine et al. 2007), Membrane Bioreactors (MBR) (Atasoy et al. 2007; Santasmasas et al. 2013), and other chemical methods (Li et al. 2009; Nolde 2005). An integrated fixed-film activated sludge (IFAS) system is one of the most popular modified activated sludge processes which increases the microbial population and accelerates the biodegradation of organic compounds by adding a fixed media to a suspended growth basin (Kim et al. 2010). This process is actually an integration process which includes the suspended and attached growth and provides the advantages of both attached and suspended growth systems (Mehrdadi et al. 2007). The IFAS process has many advantages in comparison with conventional processes of the activated sludge. This system provides more resistance against organic and hydraulic shock load; besides, it has more flexibility and higher efficacy than other activated sludge processes (Regmi et al. 2011; Rosso et al. 2011). The IFAS system is a good option to upgrade the Activated Sludge System especially in case of facing scarcity of land and provides higher removal efficiency of COD and nutrients relative to conventional activated sludge. It also possesses a lower retention time, higher hydraulic load, and less tank volume (Andreottola et al. 2003; Eslami et al. 2017b). Furthermore, this system is used to increase the removal efficiency of nitrogen and phosphorus as well (Rosso et al. 2011).

Finally, due to drought and lack of water resources in Iran and the importance of separation, treatment, and reuse of greywater, this study aimed to determine the performance of IFAS system in treatment and nutrients removal from the raw greywater.

## Materials and methods

### IFAS pilot plant setup

**Fig. 1:**
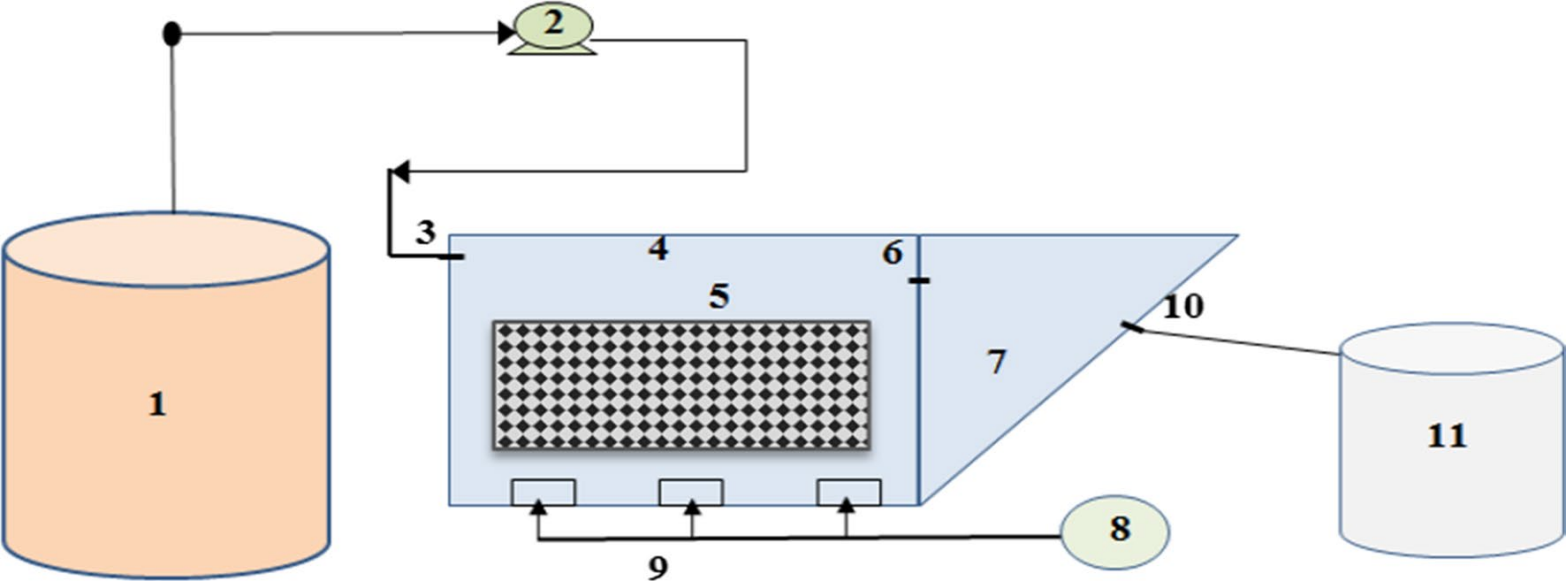
Scheme of IFAS pilot system (1 Feed tank, 2 injection pumps, 3 input of IFAS aeration reactor, 4 aerated reactor, 5 media, 6 aeration reactor outlet, 7 settling basin, 8 air pump, 9 air inlet pipes, 10 effluent, 11 effluent storage tank)

Schematic of IFAS process is shown in Fig. 1. IFAS system was composed of transparent Plexiglas sheets with a thickness of 6 mm, an aeration basin, and a sedimentation unit. The aeration basin was rectangular with length, width, and height of 30, 20, and 15 cm, respectively, and was designed with free height of 5 cm and useful volume of 9 L. The fixed media used in the aeration unit was made of PVC and had honeycomb pattern with a specific surface of 350 m^2^/m^3^ occupying 25% of the aeration reactor volume. The length, width, and height of the sedimentation unit in IFAS system were 20 cm at the surface, 10 cm at the floor, and 15 cm, respectively. Temperature control was done by a heater aquarium embedded in the aeration basin at 30 ± 1 °C. Required air for the aeration reactor was supplied thorough pumping of an AIR-8000 air pump with an air flow rate of 9 L/min mounted on the floor of the reactor. Feeding the reactors was carried out through picking up greywater from the feed tank by peristaltic pumps (ETATRON Italy) with a flow rate of 20 L/day. Organic loading rate in IFAS system at 5 loadings was 0.11–1.3 gCOD/L.d, hydraulic retention time was 10.8 h, and Input COD in 5 OLRs was 50–600 mg/L according to a previously reported study (Eslami et al. 2017a).

### Raw greywater characteristics

The raw greywater characteristics are shown in Table 1. The formula for greywater was in accordance with COD 200 mg/L (Eslami et al. 2017a; Hourlier et al. 2010; Zhu et al. 2014), and also to prepare CODs of 50, 200, 300, 400, and 600 mg/L, the formula for making greywater was diluted/concentrated according to the mentioned formula.

**Table 1:** Raw greywater formula

Chemical substance	Amount per liter	Commercial products	Amount per liter (mg)
Secondary effluent	20 mL	Deodorant	10
H_3_BO_3_	1.4 mg	Shampoo	720
C_6_H_12_O_6_	28 mg	Laundry	150
Na_2_HPO_4_	39 mg	Sunscreen or moisturizer	10–15
Na_2_SO_4_	35 mg	Toothpaste	32.5
NaHCO_3_	25 mg	Vegetable oil	7
Clay (unimin)	50 mg		

### Seeding and operation of IFAS system

To set up the system, at first, the input sludge to aerobic digester of the Yazd municipal wastewater plant with the characteristics of Mixed Liquor Suspended Solids (MLSS) = 5500 mg/L, Mixed Liquor Volatile Suspended Solids (MLVSS) = 4510 mg/L, and the Sludge Volume Index (SVI) = 160 mL/gr was used. After 25 days and formation of fixed-biofilm on aeration reactor, firstly the greywater with COD 50 mg/L and OLR of 0.11 gCOD/L.d was injected into the system, and then different greywater samples with CODs of 100, 200, 400, and 600 mg/L and OLRs of 0.22, 0.44, 0.88, and 1.3 gCOD/L.d were logged in IFAS system.

### Analysis methods

In the current study, to determine the pilot efficiency of IFAS in greywater treatment during 105 days, the BOD_5_, COD, Total Suspended Solids **(**TSS**)**, Total Nitrogen (TN), and Total Phosphorous (TP) parameters as well as pH, DO, temperature, and MLSS were measured. Composite Sampling was done on input and output from the system, and at least five samples were measured for each parameter in each loading. Measurement of the parameters was carried out via diverse methods as follows: COD by dichromate method (closed reflux, 5220 B, colorimetric method, and Spectrophotometer Milton Roy Company 2OD), BOD_5_ by Winkler method with standard Number of 2510, TSS by spectrophotometry with standard Number of 2540 D, total nitrogen (TN) by the Kjeldahl method, total phosphorus (TP) by digestion and Conversion of the various forms of phosphorus to orthophosphate, and determination of the orthophosphate by spectrophotometry. Also, temperature, pH, and DO were measured by portable YSI and MLSS using filter paper of 0.45 micron according to the standard (APHA 2005). Moreover, Scanning Electron Microscopy (SEM) (TESCAN VEGA3, Czech Republic) was used to prepare images from biofilm. In this study, mean and standard deviation of parameters as well as one-way analysis of variance (ANOVA) test were used for comparison of the removal efficiencies at different loadings for each parameter.

## Results

### SEM images

**Fig. 2:**
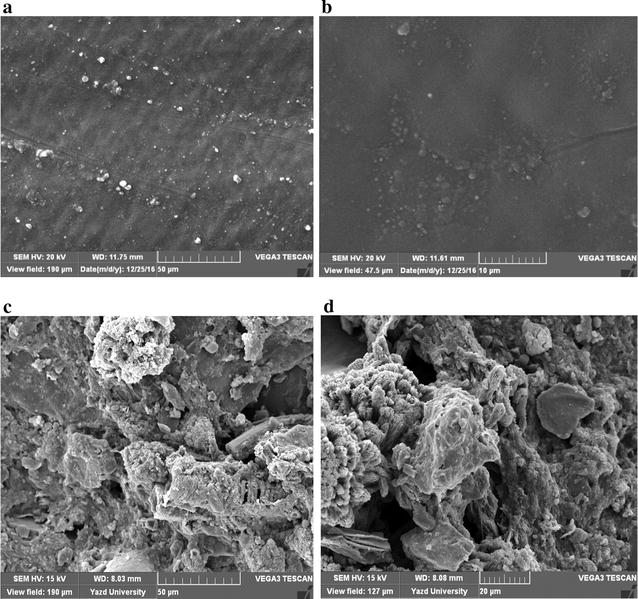
SEM images from media within IFAS reactor, (**a**, **b**) before biofilm formation, (**c**, **d**) after biofilm formation

Figure 2 shows the SEM images before and after biofilm formation on the surface media in the IFAS reactor. As seen, the biofilm after seeding was formed on the media. For sampling of biofilm, samples were dried at first and then transferred to the SEM lab. Table 2 presents the different parameters’ specifications in the greywater incoming to IFAS system at diverse OLRs. The mean pH value was 8.01 ± 95 at all organic loadings.

**Table 2:** Mean and standard deviation of BOD_5_, COD and TSS parameters in the greywater incoming to the system with diverse OLRs

Operation time (day)	OLR (mgCOD/L.d)	BOD_5_ (mg/L)	COD (mg/L)	TSS (mg/L)
25–37	111.11	15.43 ± 3.1	55.23 ± 7.1	7.11 ± 1.2
38–47	222.2	35.52 ± 8.6	97.33 ± 8.3	15.37 ± 1.8
48–67	444.4	75.34 ± 9.7	205.12 ± 10.2	30.21 ± 3.9
68–85	888.8	110.48 ± 10.4	401.44 ± 12.8	46.13 ± 8.1
86–105	1333.3	174.33 ± 12.1	610.48 ± 18.2	62.66 ± 9.5

### BOD_5_, COD and TSS removal

**Fig. 3:**
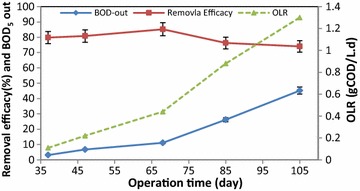
Mean changes and removal efficiency of BOD_5_ at different OLRs over the operation time

**Fig. 4:**
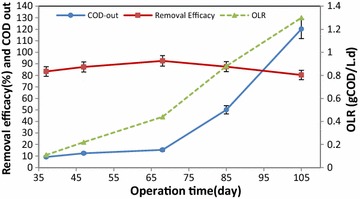
Removal efficiency and mean changes of COD at different OLRs during their operation time

**Fig. 5:**
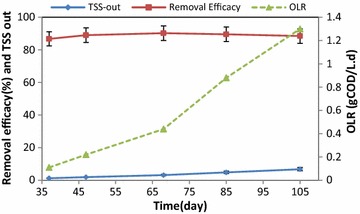
Mean changes in TSS removal efficiency at different OLRs over the operation

Figure 3 shows the output and removal efficiency of BOD_5_ at different OLRs during the 105 days of the operation. The best removal efficiency of BOD_5_ was at an OLR of 0.44 gCOD/L.d at the rate of 85.24 ± 3.21%, while the greywater entered into the system with no dilution at this stage. Figure 4 shows the efficiency of IFAS system in terms of COD removal. As it can be seen, the best removal efficiency of COD was 92.52 ± 4.33% at an OLR of 0.44 gCOD/L.d. Figure 5 presents the results of IFAS system efficiency for TSS removal from greywater at different OLRs. The best removal of TSS was 90.22 ± 3.21% obtained at an OLR of 0.44 gCOD/L.d. ANOVA test results showed that the difference between removal efficiencies in diverse loadings was insignificant (p = 0.23).

### Nutrient removal

**Fig. 6:**
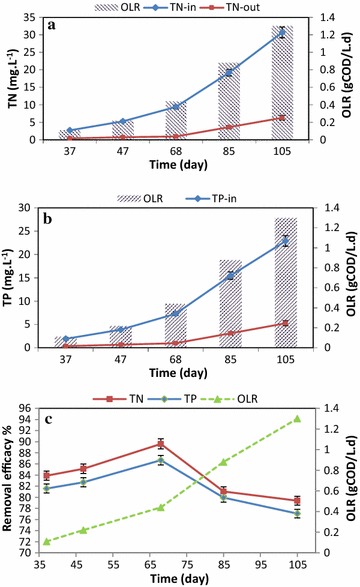
Mean and standard deviation of TN (**a**) and TP (**b**) in the inlet and outlet of IFAS system at different OLRs. **c** Removal of nutrients at different OLRs

In Fig. 6, the mean changes of input and output of TN (Fig. 6a) and TP (Fig. 6b) in IFAS reactor are shown regarding different OLRs, while Fig. 6c shows the mean removal efficiency. As shown, the best efficiencies of TN and TP were 89.60 ± 2.41 and 86.67 ± 2.14%, respectively, at an OLR of 0.44 gCOD/L.d. Also, by increasing OLR to 0.44 gCOD/L.d, removal efficiencies of TN and TP increased, but with the OLR increased more, the removal efficiency decreased. The ANOVA test revealed that the difference between removal efficiencies at diverse OLRs was statistically significant (p = 0.021).

### Relationship between the MLSS and OLR

**Fig. 7:**
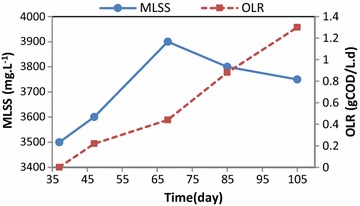
Mean changes of MLSS at different OLRs over the operation time

The mean MLSS changes at different OLRs over the operation are shown in Fig. 7. As depicted, the maximum MLSS was 3900 mg/L at an OLR of 0.44 gCOD/L.d.

## Discussion

In the present study, the best removal efficiency of BOD_5_ was 85.24%. A study by Abdel-Shafy et al. (2014) on greywater treatment using a hybrid system showed that the removal efficiency for BOD_5_ was 70.6%. Do Couto et al. (2015) studied greywater treatment via anaerobic filter system with UV disinfection. Their results revealed that BOD_5_ removal efficiency in the output filter was 73%. Saumya et al. (2015) used a constructed wetland system with a *Heliconia angusta* plant for raw greywater treatment, so removal efficiency of BOD_5_ was 70%. In a study by Abdel-Kader (2013) on the treatment of greywater by the RBC process and disinfection with UV light, it was shown that the removal efficiency of BOD_5_ was 93–96%; furthermore, in a study by Hourlier et al. (2010), the BOD_5_ removal efficiency from synthetic greywater was lower than that in real greywater. Therefore, we could conclude that synthetic greywater had low biodegradability capability as compared to real ‎greywater‎, and its removal values were lower than those of real greywater. Moreover, aerobic systems showed better performance in the removal of BOD_5_ from greywater relative to anaerobic systems which might be due to the presence of surfactants in the input greywater and their the negative effects on anaerobic microorganisms (Ghunmi et al. 2010; Hernández Leal et al. 2010). As exhibited in Fig. 2, increase of OLR caused increase of BOD removal which reached its maximum value at an OLR of 0.44 gCOD/L.d. Next, with the OLR increased more, efficiency decreased. Consequently, ANOVA statistical test results showed a significant difference between the removal efficiencies at different OLRs (p ≤ 0.001). By increasing the contact time of the greywater with microorganisms and allowing longer adaption, the removal efficiency also enhanced. Therefore, by increasing input BOD_5_ and supplying more organic materials for microorganisms, the removal efficiency soared. But at OLRs of 0.88 and 1.3 gCOD/L.d, the removal efficiency decreased, so it could be argued that higher levels of BOD_5_ in greywater, due to the xenobiotic substances, led to decrease of removal efficiency (Jabornig and Favero 2013).

The best removal efficiency of COD was 92.52%, and by increasing OLR in the loadings higher than 0.44 gCOD/L.d, removal efficiency of COD decreased. Increasing the amount of non-biodegradable substances could be related to the effects of increasing the amount of xenobiotic substances in greywater and subsequent reduction of microbial population which reduced the system efficiency at high OLRs. Comparing the removal of BOD_5_ and COD indicated that the system was more efficient in removal of COD as compared to BOD_5_, while the mean removal was higher than that of similar studies with removal efficiencies of 60–80% (Abdel-Shafy et al. 2014; Friedler et al. 2005; Jabornig and Favero 2013). Comparison of the results showed that the IFAS system had a better performance than other systems due to its integrated system of attached and suspended growth. In fact, a collection of microorganisms with attached and suspended growth could increase the removal efficiency of organic materials (Mahendran et al. 2012). The higher COD removal efficiency relative to BOD_5_ could be related to the bio sorption, adsorption, and sedimentation of non-biodegradable substances in the greywater on the biofilm and media in IFAS reactor (Eslami et al. 2017a; Nabizadeh et al. 2008).

The best removal efficiency of TSS was 90.22%. In a study by Friedler et al. (2005), the TSS removal efficiency in greywater was 82%. On the other hand, Abdel-Shafy et al. (2014) reported removal efficiency of TSS 70.8%. Furthermore, in a study by Saumya et al. (2015), the removal efficiency of TSS was 61.65%. Comparing the results of different studies with those of the present study revealed that the IFAS system has had high efficiency in TSS removal which might be due to the fixed media of the biological reactor. Indeed, hybrid systems like IFAS create the conditions of attached and suspended biological growth, and thereby the sludge sedimentation capability improves, so suspended solids along with the sludge are deposited faster with better conditions (Kim et al. 2010). The obtained results were in line with those of Jabornig and Favero (2013) who used Moving Bed Biofilm Membrane Reactor (MBBMR) system for treatment of greywater, while their reported TSS removal efficiency was 98%.

The best removal efficiencies of TN and TP were 89.6 and 86.67%, respectively. Previous studies have showed that IFAS system has a relative efficiency for removal of nutrients from wastewater. Regmi et al. (2011) examined the removal of nitrogen in IFAS system and showed the nitrogen removal in the range of 39–89%. Masterson et al. (2004) investigated the removal of nitrogen in IFAS system and showed the suitability of this system for removal of nutrients since it could reduce nitrogen in the effluent to 5 mg/L. In a study by Zhang et al. (2015), the nitrogen removal efficiency in IFAS system was 80%. Bai et al. (2016) showed that the efficiency of IFAS system for removal of TN and TP was 70 and 81%, respectively. Proper operation of IFAS system for removal of TN can be related to the further growth of nitrification and de-nitrification bacteria on the surface of the biofilm created on media as compared to conventional activated sludge process (Bai et al. 2016; Uddin et al. 2016). Moreover, the suitable phosphorus removal efficiency in IFAS system can be linked to the bacteria in suspension and on the surface of a biofilm which requires phosphorus for its growth (Uddin et al. 2016) or can be related to Phosphorus Accumulating Organisms (PAOs) which mostly tend to have suspended growth (Bai et al. 2016). Also, it can be related to the density and the high settling of sludge in IFAS system in comparison with conventional activated sludge process (Kim et al. 2010). In the present study, by increasing OLR to 0.44 gCOD/L.d, removal efficiencies of TN and TP increased, yet with the OLR increased more, the removal efficiency decreased. Due to the low needed oxygen and the low number of microorganisms at high loadings, nitrification and de-nitrification were limited, and the nitrogen removal decreased. In these conditions, the amount of MLSS and sludge formation also reduced and caused reduction in phosphorus removal (Uddin et al. 2016).

The maximum MLSS occurred at an OLR of 0.44 gCOD/L.d. According to the best efficiency of BOD_5_, COD, and TSS at this OLR, it could be concluded that the large population of the bacteria played an important role in greywater treatment. The MLSS was declined by increasing of OLR greater than 0.44 gCOD/L.d and COD greater than 200 mg/L. The reason could be the adverse effect of the high amounts of OLR and subsequently the impact of xenobiotic and non-biodegradable compositions on the microbial population which led to abatement of the MLSS (Eriksson et al. 2002; Liberman et al. 2016). Also, as a result of increasing of produced exopolysaccharide (EPSs) in attached growth process, suspended solids in IFAS reactor were precipitated, so MLSS decreased (Schnurr and Allen 2015).

In the present study, the efficiency of IFAS system in synthetic greywater treatment from different organic loadings’ rates was studied within a duration of 105 days. SEM image showed that the biofilm was formed on the media of IFAS reactor. The best removal efficiencies of BOD_5_, COD, and TSS were 85.24, 92.52 and 90.21%, respectively, at an OLR of 0.44 gCOD/L.d. Then, by increasing the OLR, removal efficiencies of BOD_5_, COD, and TSS decreased, and this reduction was statistically significant for BOD_5_ and COD, while it was insignificant for TSS. The efficiency of the IFAS system in terms of removal of nutrients showed that the best efficiencies of TN and TP were 89.60 and 86.67%, respectively, at an OLR of 0.44 gCOD/L.d. By increasing the OLR up to 0.44 gCOD/L.d, TN and TP removal efficiencies increased; then, the removal efficiencies decreased with the OLR increasing, and the difference between removal efficiencies in different OLR was statistically significant (p = 0.021). The highest MLSS was 3900 mg/L at the OLR of 0.44 gCOD/L.d. The results showed that the IFAS system provides a high efficiency in synthetic greywater treatment.

## Abbreviations

IFAS: integrated fixed-film activated sludge
OLRs: organic loadings rates
SEM: scanning electron microscope
TN: total nitrogen
TP: total phosphorus
NTU: nephelometric turbidity unit
UASB: up-flow anaerobic sludge blanket
RBC: rotating biological contactor
SBR: sequencing batch reactor
MBR: membrane bioreactor
MLSS: mixed liquor suspended solids
MLVSS: mixed liquor volatile suspended solids
SVI: sludge volume index
TSS: total suspended solids
ANOVA: one-way analysis of variance
